# OnabotulinumtoxinA injections for the pain relief and long-term symptom control in a patient with hemiplegic migraine: case study

**DOI:** 10.1186/1129-2377-14-S1-P188

**Published:** 2013-02-21

**Authors:** P Dhawan, P Dhawan

**Affiliations:** 1 Dhawan Medical Corporation, Canada

## Introduction/background

Treatment of Hemiplegic Migraine can be challenging. Migraine-specific abortives, the triptans and ergotamines, are currently contraindicated in the treatment of Hemiplegic Migraine because of their vasoconstrictive properties.

## Method

Case study to report the effects of OnabotulinumtoxinA injections for the pain relief and long-term symptom control in a patient with Hemiplegic Migraine. Case: 54-year-old woman with Hemiplegic Migraine for the past 12 years. Severe migraines with right unilateral retro-orbital pain, with progression to the left side, followed by right facial weakness with complete ptosis on the right side and less prominent left facial weakness, immediately progressing to right hemiparesis and then later to left hemiparesis. Attacks were accompanied with nausea, vomiting, dysarthria and dysphasia. Migraine and paralytic episodes 1/week lasting 3-5 days. Headache frequency 20 days/month. Failed the following therapy: Topamax, Epival and Memantine. Inconsistently responded to IV Maxeran and Benadryl in ER. First OnabotulinumtoxinA injections received in September, 2009. 100 units of the toxin injected over frontalis, corrugators, procerus, temporalis, occipitalis, sub-occipital and splenius capitus bilaterally. Outcome measures: Frequency of migraine and pain intensity (mild, moderate, severe) and limb weakness was measured at baseline and at 90 days after treatment.

## Results

Immediate and sustained response since first treatment cycle. Frequency of Migraine episodes with paralysis decreased from 12 to 2-3 episodes in 3 months. Migraine severity decreased from severe to mild. Not a single ER visit since! Mild facial weakness only during the attacks. Migraine aborted by self administration of IM Maxeran and Benadryl 2-3 times/3 months between injections. Also on 100 mg of Amitriptylin all along. Effect of OnabotulinumtoxinA wears off in 3 months. No side effects reported.

## Conclusion

OnabotulinumtoxinA appears to help in alleviating symptoms and reduce care needs of Hemiplegic Migraine in this case.

## Conflict of interest

None.

## References

[B1] Aurora SK Dodick DW "OnabotulinumtoxinA for treatment of chronic migraine: Results from the double-blind, randomized, placebo-controlled phase of the PREEMPT 1 trial" Cephalgia OnlineFirst 2010 doi:10.1177/0333102410364676 20647170 10.1177/0333102410364676

[B2] Diener HC Dodick DW "OnabotulinumtoxinA for treatment of chronic migraine: Results from the double-blind, randomized, placebo-controlled phase of the PREEMPT 2 trial" Cephalgia OnlineFirst 2010 doi:10.1177/0333102410364677 20647171 10.1177/0333102410364677

